# 1995. Duration of letermovir primary prophylaxis in hematopoietic cell transplant recipients at high risk for cytomegalovirus infections and impact on outcomes

**DOI:** 10.1093/ofid/ofad500.122

**Published:** 2023-11-27

**Authors:** Anthony J Febres-Aldana, Fareed Khawaja, Amy Spallone, Krithika Srinivasan, Terri Lynn Shigle, Oscar Morado Aramburo, Gabriella Rondon, Jeremy Ramdial, Elizabeth Shpall, Ella Ariza Heredia, Roy F Chemaly, Joseph Sassine

**Affiliations:** MD Anderson Cancer Center, Wesley Chapel, FL; The University of Texas MD Anderson Cancer Center, Houston, Texas; University of Texas MD Anderson Cancer Center, Houston, Texas; Stanford, Palo Alto, California; The University of Texas MD Anderson Cancer Center, Houston, Texas; The University of Texas MD Anderson Cancer Center, Houston, Texas; The University of Texas MD Anderson Cancer Center, Houston, Texas; MD Anderson Cancer Center, Wesley Chapel, FL; The University of Texas MD Anderson Cancer Center, Houston, Texas; The University of Texas MD Anderson Cancer Center, Houston, Texas; MD Anderson, Houston, Texas; University of Oklahoma Health Sciences Center, Oklahoma City, Oklahoma

## Abstract

**Background:**

The incidence of clinically significant cytomegalovirus infection (CS-CMVi) in high risk allo-HCT recipients who are CMV seropositive (R+) has decreased in the era of letermovir (LTV) primary prophylaxis (PP) which is also associated with a reduction in all-cause mortality (ACM) and non-relapse mortality (NRM). Most reduction of CS-CMVi occur within the first 100 days when CMV PP is employed but it is unclear if PP beyond 100 days further impacts CMV outcomes and mortality.

**Methods:**

In a single-center study of CMV R+ recipients of allo-HCT between March 2016 and December 2019, we compared the outcomes for those who were not on LTV PP to those on LTV PP for < 100 days, 100 – 200 days, and > 200 days. Data on baseline and transplant characteristics was collected in addition to the outcomes that included the incidence of CS-CMVi before and after day 100, CMV end-organ disease, and ACM and NRM at day 100 and week 48. Univariate analysis was done with Fisher’s exact test or Wilcoxon rank sum test. Survival curves were constructed using landmark times of day 100 and 200. Patients who relapsed after transplant were excluded from NRM analyses.

**Results:**

940 R+ patients underwent allo-HCT; 533 were not on LTV, 144 received LTV for < 100 days, 146 for 100-200 days, and 117 for > 200 days. The source of the HCT, the rates of ATG and post-Cy, and the choice of GVHD prophylaxis diferred among groups (Table 1). The rate of CS-CMVi was 44.5% in patients not on LTV, which decreased with LTV PP at all time points. Most cases of CS-CMVi occurred within 100 days after HCT (Table 2), with 0.84% of the cases in R+ not on LTV occurring after day 100 compared to 7.5% with LTV PP, most occurring after discontinuation of LTV. When LTV was used >200 days, 66.6% of these patients had CS-CMVi occurring as breakthrough infections. The ACM was similar among the patients, except for lower ACM at week 48 for patients on LTV > 200 days. NRM at week 48 was lower in the patients on LTV for 100-200 days. Survival curves demonstrated a higher survival of R+ recipients on LTV PP for 100 – 200 days.

**Table 1.**
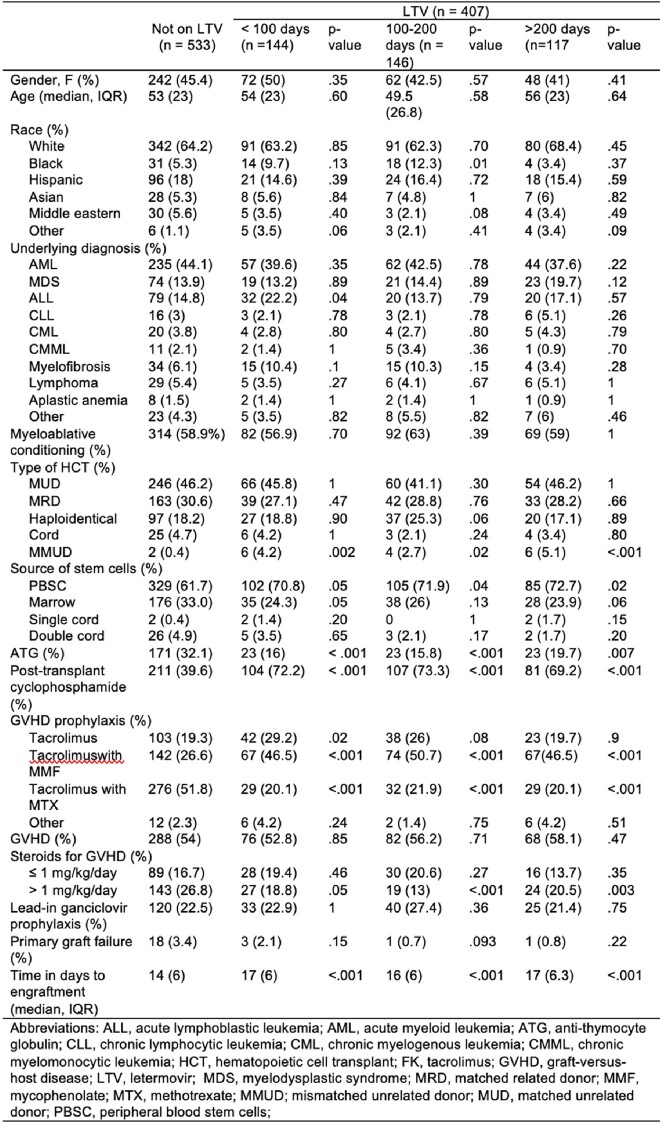
Univariate analysis of the baseline characteristics of the 940 patients of the cohort of allogenic hematopoietic cell transplant recipients.

**Table 2.**
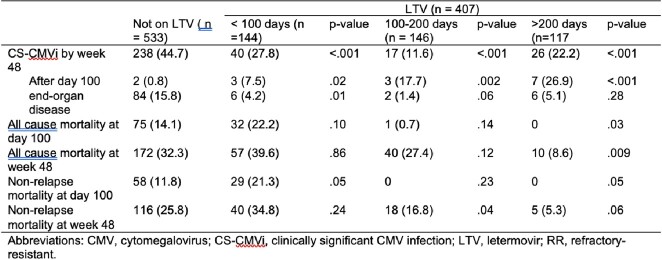
CMV related outcomes on univariate analysis

**Figure 1.**
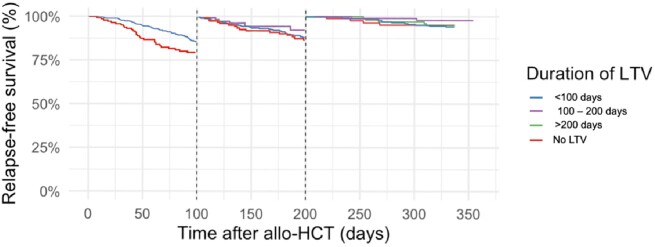
Kaplan-Meier curves depicting the patients not on LTV primary prophylaxis compared with those on LTV, establishing landmark times at days 100 and 200 after the transplant.

**Conclusion:**

Our study shows a consistent reduction in CS-CMVi and CMV end-organ disease for allo-HCT recipients while on LTV PP beyond 100 days. ACM and NRM decreased with LTV PP, with higher benefit for allo-HCT recipients on LTV PP beyond 200 days.

**Disclosures:**

**Fareed Khawaja, MBBS**, MEDSCAPE: Honoraria|Viracor: Grant/Research Support **Terri Lynn Shigle, PharmD, BCOP**, Takeda: Advisor/Consultant **Gabriella Rondon, MD**, Omeros: Advisor/Consultant **Elizabeth Shpall, MD**, Adaptimmune: Advisor/Consultant|Axio: Advisor/Consultant|Bayer Healthcare Pharmaceuticals: Honoraria|Fibroblast and FibrioBiologics: Advisor/Consultant|Navan: Advisor/Consultant **Ella Ariza Heredia, MD**, Merck: Grant/Research Support **Roy F. Chemaly, MD/MPH**, Eurofins-VViracor: Grant/Research Support|Karius: Advisor/Consultant

